# Discovery of potential biomarkers for osteoporosis using LC/GC−MS metabolomic methods

**DOI:** 10.3389/fendo.2023.1332216

**Published:** 2024-01-17

**Authors:** Yahui Wu, Chunhua Yuan, Peipei Han, Jiangling Guo, Yue Wang, Cheng Chen, Chuanjun Huang, Kai Zheng, Yiqiong Qi, Jiajin Li, Zhengjie Xue, Fanchen Lu, Dongyu Liang, Jing Gao, Xingyan Li, Qi Guo

**Affiliations:** ^1^ School of Health Science and Engineering, University of Shanghai for Science and Technology, Shanghai, China; ^2^ Department of Rehabilitation Medicine, Shanghai University of Medicine and Health Sciences Affiliated Zhoupu Hospital, Shanghai, China; ^3^ Comprehensive surgical rehabilitation ward, Shanghai Health Rehabilitation Hospital, Shanghai, China; ^4^ Graduate School of Shanghai University of Traditional Chinese Medicine, Shanghai University of Traditional Chinese Medicine, Shanghai, China; ^5^ School of Health, Fujian Medical University, Fujian, China; ^6^ Department of Sport Rehabilitation, Shanghai University of Sport, Shanghai, China; ^7^ Clinical Research Center, Jiading District Central Hospital Affiliated Shanghai University of Medicine & Health Sciences, Shanghai, China; ^8^ General Practice Clinic, Pujiang Community Health Service Center in Minhang District, Shanghai, China; ^9^ Shanghai Hongkou District Jiangwan Hospital Affiliated Shanghai University of Medicine & Health Sciences, Shanghai, China

**Keywords:** untargeted metabolomics, osteoporosis, liquid chromatography, gas chromatography, mass spectrometry, biomarkers

## Abstract

**Purpose:**

For early diagnosis of osteoporosis (OP), plasma metabolomics of OP was studied by untargeted LC/GC−MS in a Chinese elderly population to find possible diagnostic biomarkers.

**Methods:**

A total of 379 Chinese community-dwelling older adults aged ≥65 years were recruited for this study. The BMD of the calcaneus was measured using quantitative ultrasound (QUS), and a T value ≤-2.5 was defined as OP. Twenty-nine men and 47 women with OP were screened, and 29 men and 36 women were matched according to age and BMI as normal controls using propensity matching. Plasma from these participants was first analyzed by untargeted LC/GC−MS, followed by FC and P values to screen for differential metabolites and heatmaps and box plots to differentiate metabolites between groups. Finally, metabolic pathway enrichment analysis of differential metabolites was performed based on KEGG, and pathways with P ≤ 0.05 were selected as enrichment pathways.

**Results:**

We screened metabolites with FC>1.2 or FC<1/1.2 and P<0.05 and found 33 differential metabolites in elderly men and 30 differential metabolites in elderly women that could be potential biomarkers for OP. 2-Aminomuconic acid semialdehyde (AUC=0.72, 95% CI 0.582-0.857, P=0.004) is highly likely to be a biomarker for screening OP in older men. Tetradecanedioic acid (AUC=0.70, 95% CI 0.575-0.818, P=0.004) is highly likely to be a biomarker for screening OP in older women.

**Conclusion:**

These findings can be applied to clinical work through further validation studies. This study also shows that metabolomic analysis has great potential for application in the early diagnosis and recurrence monitoring of OP in elderly individuals.

## 1 Introduction

Osteoporosis (OP) is a systemic disease characterized by decreased bone mass and microstructural destruction of bone tissue, resulting in increased bone fragility and susceptibility to fracture (1). A survey conducted by the World Health Organization indicates that the current prevalence of OP exceeds 200 million individuals, with a continuous annual rise in incidence (2). In China, OP afflicts over one-third of individuals aged 50 years and older (3). OP has emerged as a significant health concern owing to its elevated prevalence, morbidity, mortality rates, and associated medical expenditures (4–7).Therefore, prevention and early detection of OP are essential for people to maintain bone health and improve their overall quality of life.

Bone mineral density (BMD) stands as the gold standard for diagnosing OP (8). Commonly employed methods for diagnosis and efficacy monitoring encompass dual-energy X-ray absorptiometry (DXA) and the assessment of bone turnover biochemical markers (BTM). However, the absence of a definitive marker poses a diagnostic challenge. Consequently, there arises an imperative to unearth novel diagnostic markers during the early phases of OP. Metabolomic measurements offer a potential avenue to unveil biomarkers for disease diagnosis or prognosis across diverse organisms, thereby significantly advancing our comprehension of the pathophysiological processes governing disease progression (9). Positioned as a metabolic disorder of bone tissue, OP exhibits a close association with metabolomics (10). Predominantly, investigations have focused on analyzing serum and plasma samples from OP patients utilizing NMR (11, 12), LC−MS (13, 14) and GC−MS (15);however, the scope has been limited to postmenopausal women. An additional study utilized LC−MS to conduct a metabolomic analysis of sera from OP patients, encompassing both women and men (10). Uniformly, these studies adopted a singular method; the identification of metabolites was relatively restricted, and the majority of investigations encompassed limited patient cohorts. Despite Kou et al.’s study employing both LC−MS and GC−MS methods, the findings were exclusively relevant to postmenopausal women, not extending to men (16). These studies’ findings were subject to specific limitations. Liquid chromatography is suitable for polar and relatively polar compounds, while gas chromatography is suitable for relatively non-polar compounds. The combination of both techniques can offer a more comprehensive and accurate compound analysis. In comparison to the standalone use of LC-MS, the combined application of LC-MS and GC-MS allows for a clearer differentiation of compounds, thereby enhancing the accuracy of analytical results.

Hence, through the integration of LC−MS and GC−MS technologies, we aim to leverage their respective technical merits for a more thorough and precise exploration of the metabolomics in both male and female osteoporosis (OP) patients. Our aspiration is to provide a substantial contribution to the early diagnosis of OP in the elderly.

## 2 Materials and methods

### 2.1 Participants

The study cohort comprised individuals aged 65 years and older, drawn from participants in the Shanghai national free physical examination program. A total of 379 subjects were extended invitations to partake in a thorough geriatric assessment, along with face-to-face interviews conducted at local community hospitals. Comprehensive details regarding the questionnaire employed have been expounded in a preceding publication (17). Ethical approval was granted by the Institutional Review Boards, and written informed consent was duly acquired from all participants.

### 2.2 Measures of osteoporosis

BMD was measured at the calcaneus by quantitative ultrasound (QUS; OsteoPro UBD2002A, BMTECH; Worldwide Co. Ltd., Seoul, Korea) (18), utilizing T scores based on World Health Organization (WHO) criteria (19), which were obtained from automated equipment. A T score ≤ -2.5 was considered to indicate OP.

### 2.3 Sample collection and processing

Out of 379 participants, we specifically categorized 29 males with OP and 47 females with OP. Employing propensity matching, we selected 29 normal male subjects and 36 normal female subjects, ensuring age and body mass index (BMI) alignment for each gender group.

### 2.4 LC−MS pretreatment method

Plasma samples were collected from fasting individuals in the morning, separated, and stored at -80°C in EDTA tubes before analysis. Before running, samples were thawed at room temperature. Following the collection of a 150 μl plasma sample, a fresh tube was utilized to combine the sample with 10 μl of L-2-chlorophenylalanine (0.3 mg/ml), dissolved in methanol and used as an internal standard. Subsequently, a 450 μl mixture of acetonitrile/methanol (1/2) was added, followed by vortexing for 1 min. The sample was extracted via ultrasound, stored at -20°C for 30 min, and then centrifuged at 13,000 rpm for 10 min at 4°C. A 200-μl sample was subjected to freeze-drying, followed by resolubilization in 300-μl methanol/water (1/4) and vortexing for 30 s. The resultant mixture was then subjected to ultrasonic extraction for 3 min. After being vigorously mixed, the samples were then centrifuged at 4°C and 13,000 RPM for 10 min. After centrifugation, 150 μl of supernatant was filtered through 0.22 μm microfilters and transferred to LC vials.

### 2.5 GC−MS pretreatment method

In the process described, a total of 150 μl sample and 20-μl of 0.3 mg/mL 2-chloro-L-phenylalanine in methanol, acting as an internal standard, were combined within a 1.5 mL Eppendorf tube. Subsequently, the mixture was subjected to vortexing for 10 s. Next, a mixture of 450 µl acetonitrile and methanol (1/2) was added. The mixture was vortexed for 30 s and the entire sample was then extracted for 10 min using ultrasound in an ice water bath. Finally, the sample was stored at -20°C for 30 min. The extract was centrifuged for 10 min (4°C 13,000 RPM). The 200 µL of supernatant was transferred to a glass bottle and dried in a freeze-concentration centrifugal dryer. Subsequently, the glass vial was filled with 80 ml of 15 mg/ml methoxyamine hydrochloride in pyridine. The ingredients were thoroughly mixed for 2 min and subsequently allowed to incubate at 37°C for 90 min for silane derivatization. Following the incubation period, a mixture of 50 μl of BSTFA (with 1% TMCS) and 20 μl of hexane was added to the mixture, which was then vigorously rotated for 2 min. The mixture underwent derivatization for 60 min at 70°C. Before conducting GC-MS analysis, the samples were left at room temperature for 30 min.

### 2.6 Metabolite measurement

Utilizing a liquid mass spectrometer system, the analytical instrument comprised an ACQUITY ultra-performance liquid chromatography (UPLC) I-Class tandem VION IMS QT of high-resolution mass spectrometer (Waters Corporation, Milford, USA). Separation of samples occurred on the ACQUITY UPLC BEH C18 column (Waters Corporation; 1.7µm, 100 × 2.1 mm) with a flow rate of 0.4 ml/min. The column temperature was maintained at 45°C, and the injection volume was set at 1 µl, while the sample chamber was held at 4°C. Mobile phases included water with 0.1% formic acid (solution A) and acetonitrile/methanol (2/3, vol/vol) with 0.1% formic acid (solution B).Linear gradient: 0min,1%B; 1min,30%B; 2.5min,60%B; 6.5min,90%B; 8.5min,100%B; 10.7min, 100% B; 10.8 min, 1% B and 13min, 1%B. Employing electrospray ionization (ESI) as the ion source, positive and negative ion scanning modes were used for sample mass spectrometry signal acquisition. For LC-MS analysis, mass spectrometric tuning parameters adopted optimized settings: ion source temperature at 150°C, capillary voltages set at 2.5 kV, desolvation gas flow at 900 L/h, declustering potential at 40 V, collision energy at 4 eV, mass scan range from m/z 50 to 1,000, and a scan time of 0.2 s (20).

An Agilent 7890B gas chromatography system linked with an Agilent 5977A MSD system (Agilent Technologies Inc., CA, USA) was employed for the analysis of derivatized samples. To separate the derivatives, a DB-5MS fused-silica capillary column (30 m x 0.25 mm x 0.25 μm, Agilent J&W Scientific, Folsom, CA, USA) was utilized. Maintaining a constant flow rate of 1 mL/min through the column, helium (>99.999%) functioned as the carrier gas. In splitless mode, the injector temperature was held at 260°C, and the injection volume was set at 1μL.The initial oven temperature commenced at 60°C and increased to 125°C at a rate of 8°C/min, followed by a ramp to 210 °C at a rate of 8°C/min, further ramping to 270°C at a rate of 15°C/min, and ultimately reaching 305°C at a rate of 20°C/min, where it was held for 5 min. MS conditions include an electron impact ionization source with an ion source temperature of 230°C and a quadrupole temperature of 150°C. The electron energy is set at 70 eV. The scanning mode is set to full scan, with a mass scan range of m/z 50-500.Throughout the analytical run, quality control samples (QCs) were injected at regular intervals (every 10 samples) to generate a dataset for assessing repeatability (21).

### 2.7 Data processing and data analysis

The LC-MS data were analyzed using Progenesis QI version 2.3 software (Nonlinear, Dynamics, Newcastle, UK) for peak alignment, peak picking, normalization, and retention time (RT) correction for data mining. The features matrix that was obtained contained data on mass-to-charge ratio (m/z), RT, and peak intensities. Metabolite processing was conducted using the Progenesis QI data processing software, which leveraged precise information on the mass-to-charge ratio (m/z), secondary fragments, and isotopic distribution. This qualitative analysis was facilitated through the integration of various databases, including the Human Metabolome Database (HMDB), Lipid Maps (version 2.3), METLIN, and internally constructed databases (EMDB).

The GC-MS data were analyzed using MS-DIAL software (version 2.74) for peak detection, identification, characterization, alignment, and wave filtering. The untargeted GC-MS database from Lumingbio (LUG) was employed for metabolite characterization. This three-dimensional matrix encompasses essential information such as sample details, peak nomenclature, retention time, retention index, mass-to-charge ratio, and signal intensity associated with each substance. The signal intensities of all peaks within each sample were subjected to segmentation and subsequent normalization, following screening with a criterion of RSD>0.3. Subsequently, the data matrix was obtained through processes involving redundancy removal and peak merging.

Fold change and P values were used to screen for differential metabolites, and metabolites meeting both FC>1.2 or FC<1/1.2 and P<0.05 were considered differential metabolites. Then, the Kyoto Encyclopedia of Genes and Genomes (KEGG) was searched to find metabolic pathways related to these key metabolites, the relevant literature was reviewed to verify their pathological relationship with OP, and the screened metabolic pathway was finally drawn. Pathway enrichment analysis was performed using the KEGG ID of differential metabolites to obtain metabolic pathway enrichment results. Hypergeometric tests were applied to identify pathway entries that were significantly enriched in significantly differentially expressed metabolites compared to the whole background, which was calculated as follows:


$$P=\sum_{i=0}^{m-1}\frac{\left(\frac{M}{i}\right)\left(\frac{N-M}{n-i}\right)}{\frac{N}{n}}$$

where N is the total number of metabolites, n is the number of differentially expressed metabolites in N, M is the number of metabolites annotated to a specific pathway, and m is the number of differential metabolites annotated to a specific pathway.

### 2.8 Statistical analysis

Baseline sociodemographic characteristics between the normal and OP groups were compared using an independent *t* test for numerical variables and the chi-squared test for categorical variables. Data with a normal distribution are expressed as the mean ± SD, and categorical variables are expressed as proportions. Statistical analyses were performed using SPSS version 26.0 (SPSS Incorporation, Chicago, IL, USA), and *P*< 0.05 was considered statistically significant. Areas under the receiver‐operating characteristic (ROC) curve (AUC) were calculated to evaluate the performance of the differential metabolites.

## 3 Results

### 3.1 Characteristics of the study population

Of the 379 participants available for analysis, 29 men and 47 women met the diagnostic criteria and were defined as having OP. We matched the male OP group (n=29) and female OP group (n=36) to the male normal (N) group (n=29) and female normal (N) group (n=36), respectively, to prevent differences in metabolic levels by age, BMI and disease status. In total, 130 participants had complete clinical and metabolomic data. **Table 1** shows the demographic and clinical characteristics of the participants. There were no significant differences between the groups in terms of age distribution, BMI and disease, indicating that the subjects in each group were comparable.

**Table 1:** Baseline sociodemographic variables of the matched groups (N=130).

	Male(n=58)			Female(n=72)		
	OP (n=29)	N (n=29)	p	OP (n=36)	N (n=36)	p
Age(y)	72.90 ± 6.43	72.10 ± 5.55	0.617	72.14 ± 4.21	71.64 ± 4.99	0.647
BMI (kg/m^2^)	22.59 ± 3.53	22.41 ± 3.55	0.853	23.85 ± 3.82	24.19 ± 2.87	0.673
BMD (T score)	-3.11 ± 0.58	-0.03 ± 1.03	<0.001	-3.01 ± 0.44	-0.23 ± 0.65	<0.001
Smoking (%)	10(34.5)	9(31.0)	0.673	1(2.8)	1(2.8)	1.000
Drinking (%)	11(37.9)	11(37.9)	0.381	3(8.3)	3(8.3)	0.995
TC (mmol/l)	4.89 ± 0.82	4.45 ± 0.83	0.048	5.66 ± 1.08	5.42 ± 0.98	0.319
HDL (mmol/l)	1.41 ± 0.33	1.33 ± 0.34	0.325	1.52 ± 0.30	1.38 ± 0.36	0.080
LDL (mmol/l)	3.01 ± 0.67	2.78 ± 0.83	0.266	3.63 ± 1.04	3.48 ± 0.85	0.498
Number of diseases						
Diabetes (%)	6(20.7)	6(20.7)	1.000	7(19.4)	4(11.1)	0.326
Hypertension (%)	15(51.7)	14(48.3)	0.793	26(72.2)	26(72.2)	1.000
Hyperlipidemia (%)	2(6.9)	6(20.7)	0.128	4(11.1)	7(19.4)	0.326
Stroke (%)	6(20.7)	7(24.1)	0.753	11(30.6)	9(25.0)	0.599
Heart disease (%)	8(27.6)	4(13.8)	0.195	12(33.3)	16(44.4)	0.334

Data are presented as mean ± SD for continuous variables and n (%) for categorical variables. OP, osteoporosis; N, normal group; BMI, body mass index; BMD, bone mineral density; TC, total cholesterol; HDL, high density lipoprotein; LDL, Low density lipoprotein.

### 3.2 Metabolomic differences between male and female study groups

**Figure 1:**
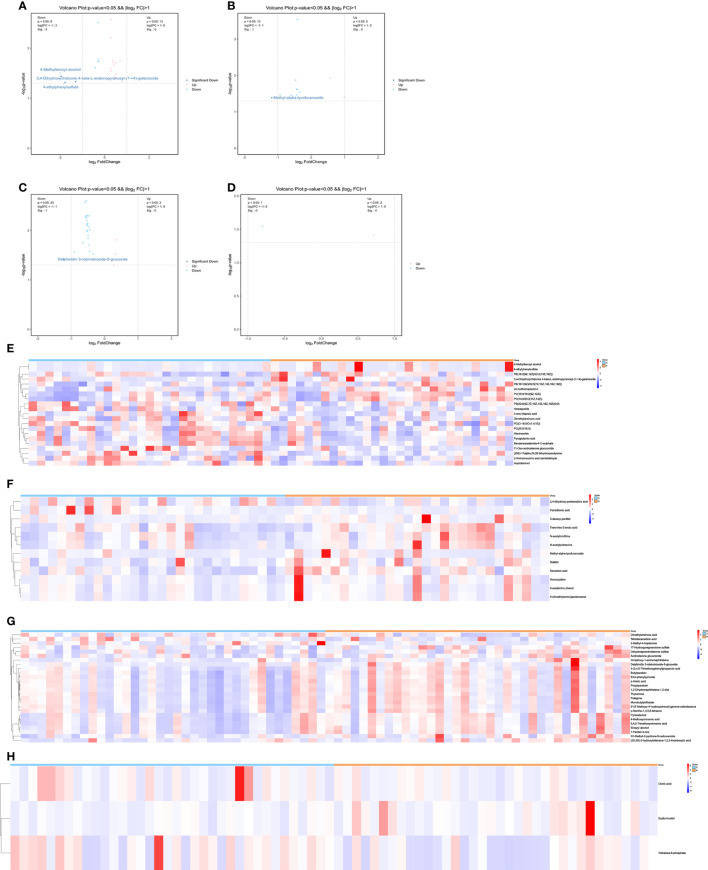
**(A)** volcano-LC(male). **(B)** volcano-GC(male). **(C)** volcano-LC(female). **(D)** volcano-GC(female). **(E)** heatmap-LC(male). **(F)** heatmap-GC(male). **(G)** heatmap-LC(female). **(H)** heatmap-GC(female).

**Figure 2:**
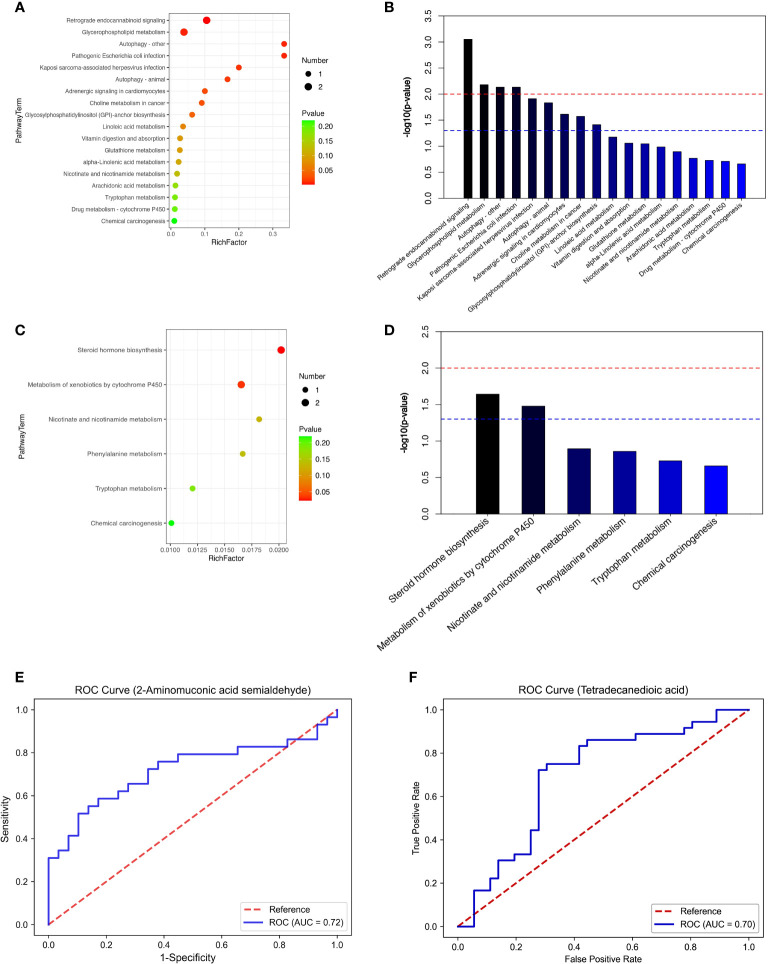
**(A)** top20-LC(male). **(B)** top20-LC(male). **(C)** top20-LC(female). **(D)** top20-LC(female). **(E)** ROC (male). **(F)** ROC (female).

We performed a comprehensive metabolomic analysis of plasma from two groups of men and women. A total of 1012 metabolites by LC−MS and 446 metabolites by GC−MS were identified in plasma. Volcano plots show the p values and fold change values (**Figures 1A–D**), thus demonstrating the validity of the differential metabolites. The levels of these metabolites were visualized by a heatmap (**Figures 1E–H**), in which colors represent increased (red) or decreased (blue) abundance, with the intensity reflecting the corresponding concentration. **Figure 1** indicates that the differences in the metabolite we chose are significant. Based on metabolites with FC>1.2 or FC<1/1.2 and P<0.05, 33 metabolites were considered potential biomarkers for OP in men, and 30 metabolites were considered potential biomarkers for OP in women (**Tables 2**, **3**). **Tables 2** and **3** show the specific metabolites designated by LC/GC−MS. Significant differences between groups can also be shown by potential biomarker box-and-whisker plots (**Supplementary Figures 1 A–E**). Among them, the levels of 18 metabolites in men and 25 in women were decreased in the OP group. In contrast, the levels of 15 metabolites in men and 5 metabolites in women were significantly increased in the OP. All the differential metabolites in the different comparison groups were matched to the KEGG database to obtain information on the pathways in which the metabolites were involved. Enrichment analysis was performed on the annotated results to obtain the pathways with high enrichment of differential metabolites. We performed pathway enrichment analysis and showed that retrograde endocannabinoid signaling, glycerophospholipid metabolism, autophagy – other, pathogenic Escherichia coli infection, steroid hormone biosynthesis, and metabolism of xenobiotics by cytochrome P450 were the most significantly perturbed pathways in OP (**Figures 2A–D**). Univariate ROC curves were analyzed for potential biomarkers and found that 2-aminomuconic acid semialdehyde (AUC=0.72, 95% CI 0.582-0.857, P=0.004) had a better diagnostic value in male OP, and Tetradecanedioic acid (AUC=0.70, 95% CI 0.575-0.818, P=0.004) had a better diagnostic value in female OP had better diagnostic value (**Figures 2E, F**).

**Table 2:** Summary of potential biomarkers of the male osteoporosis group by plasma LC/GC-MS analysis.

Metabolites	Status^a^	FC^b^	P-value^b^	VIP^c^	Data origin
Benzeneacetamide-4-O-sulphate	↑	1.723	0.046	1.258	LC-MS
11-Oxo-androsterone glucuronide	↑	1.627	0.018	0.094	LC-MS
PC(25:0/18:0)	↑	1.443	0.020	1.241	LC-MS
2-ene-Valproic acid	↑	1.422	0.033	0.237	LC-MS
Pyroglutamic acid	↑	1.419	0.040	1.142	LC-MS
Niacinamide	↑	1.374	0.038	0.710	LC-MS
(25S)-11alpha,20,26-trihydroxyecdysone	↑	1.349	0.022	0.385	LC-MS
Dimethylarsinous acid	↑	1.315	0.020	0.239	LC-MS
Harpagoside	↑	1.288	0.018	0.006	LC-MS
2-Aminomuconic acid semialdehyde	↑	1.227	0.003	0.267	LC-MS
PC(O-18:0/O-2:1(1E))	↑	1.223	0.029	0.508	LC-MS
Isoproterenol	↑	1.223	0.006	0.429	LC-MS
PS(22:6(4Z,7Z,10Z,13Z,16Z,19Z)/0:0)	↑	1.207	0.026	0.047	LC-MS
24-northornasterol A	↓	0.750	0.025	0.864	LC-MS
3,4-Dihydroxychalcone 4-beta-L-arabinopyranosyl-(1->4)-galactoside	↓	0.405	0.047	0.092	LC-MS
4-ethylphenylsulfate	↓	0.293	0.049	0.624	LC-MS
4-Methylbenzyl alcohol	↓	0.259	0.037	0.171	LC-MS
PC (15:0/18:2(9Z,12Z))	↓	0.832	0.017	5.989	LC-MS
PC (14:0/20:2(11Z,14Z))	↓	0.833	0.018	47.019	LC-MS
PE (18:2(9Z,12Z)/22:2(13Z,16Z))	↓	0.817	0.018	1.174	LC-MS
PE (16:1(9Z)/22:5(7Z,10Z,13Z,16Z,19Z))	↓	0.818	0.003	1.023	LC-MS
Pantothenic acid	↑	1.984	0.039	0.220	GC-MS
2,4-dihydroxy-pentanedioic acid	↑	1.483	0.012	0.293	GC-MS
N-acetylornithine	↓	0.791	0.029	0.140	GC-MS
N-acetylputrescine	↓	0.754	0.024	0.137	GC-MS
Senecioic acid	↓	0.754	0.000	0.153	GC-MS
Homocystine	↓	0.739	0.024	0.061	GC-MS
Maltitol	↓	0.737	0.034	0.094	GC-MS
Trans-hex-2-enoic acid	↓	0.725	0.013	0.046	GC-MS
4-acetamino phenol	↓	0.679	0.043	0.350	GC-MS
4-(dimethylamino)azobenzene	↓	0.661	0.035	0.204	GC-MS
3-desoxy-pentitol	↓	0.525	0.034	0.062	GC-MS
Methyl-alpha-lyxofuranoside	↓	0.442	0.041	0.413	GC-MS

^a^Relative concentrations compared to normal group: ↑ = upregulated, ↓ = downregulated. ^b^Fold change between OP patients and normal group ^b^ *p* value determined from Student’s t-test. ^c^Correlation coefficient and VIP value were obtained from OPLS-DA analysis.

**Table 3:** Summary of potential biomarkers of the female osteoporosis group by plasma LC/GC-MS analysis.

Metabolites	Status^a^	FC^b^	P-value^b^	VIP^c^	Data origin
2-Methyl-4-heptanone	↑	1.282	0.015	0.334	LC-MS
Dimethylarsinous acid	↑	1.254	0.030	0.282	LC-MS
Tetradecanedioic acid	↑	1.205	0.050	0.465	LC-MS
N-Hydroxy-1-aminonaphthalene	↓	0.789	0.030	0.992	LC-MS
Butylparaben	↓	0.734	0.010	0.729	LC-MS
Androsterone glucuronide	↓	0.732	0.027	0.653	LC-MS
Enol-phenylpyruvate	↓	0.725	0.008	0.686	LC-MS
Dehydroepiandrosterone sulfate	↓	0.723	0.020	8.282	LC-MS
17-Hydroxypregnenolone sulfate	↓	0.722	0.005	0.716	LC-MS
p-Anisic acid	↓	0.711	0.015	0.681	LC-MS
1,2-Dihydronaphthalene-1,2-diol	↓	0.710	0.013	0.628	LC-MS
Monobutylphthalate	↓	0.704	0.007	0.936	LC-MS
Thysanone	↓	0.703	0.010	0.537	LC-MS
Pulegone	↓	0.702	0.008	2.056	LC-MS
4-Methoxycinnamic acid	↓	0.697	0.005	0.837	LC-MS
3,4,5-Trimethoxycinnamic acid	↓	0.693	0.006	2.324	LC-MS
5’-(3’-Methoxy-4’-hydroxyphenyl)-gamma-valerolactone	↓	0.693	0.008	2.091	LC-MS
p-Mentha-1,3,5,8-tetraene	↓	0.690	0.010	0.419	LC-MS
1-Penten-3-one	↓	0.685	0.006	0.592	LC-MS
N1-Methyl-2-pyridone-5-carboxamide	↓	0.685	0.003	0.978	LC-MS
Propylparaben	↓	0.685	0.005	5.981	LC-MS
Sinapyl alcohol	↓	0.676	0.005	2.880	LC-MS
Pyrocatechol	↓	0.668	0.003	0.676	LC-MS
3-(3,4,5-Trimethoxyphenyl)propanoic acid	↓	0.638	0.018	0.569	LC-MS
(2S,3S)-2-hydroxytridecane-1,2,3-tricarboxylic acid	↓	0.535	0.028	0.490	LC-MS
Delphinidin 3-robinobioside-5-glucoside	↓	0.431	0.038	0.698	LC-MS
11-beta-Hydroxyandrosterone-3-glucuronide	↓	0.803	0.015	0.558	LC-MS
Cholic acid	↑	1.645	0.039	0.064	GC-MS
Trehalose-6-phosphate	↑	1.644	0.012	0.122	GC-MS
Scyllo-inositol	↓	0.574	0.028	0.107	GC-MS

a Relative concentrations compared to normal group: ↑ = upregulated, ↓ = downregulated.

b Fold change between OP patients and normal group. *p* value determined from Student’s t-test.

c Correlation coefficient and VIP value were obtained from OPLS-DA analysis.

## 4 Discussion

In this investigation, an untargeted LC/GC−MS metabolomics approach was deployed to scrutinize plasma metabolism attributes in elderly individuals of both genders diagnosed with osteoporosis (OP). Our data analysis revealed a constellation of metabolites intricately linked with OP, particularly delineating 18 pertinent metabolic pathways in men and 6 in women. Notably, the most prominent metabolic pathways encompassed retrograde endocannabinoid signaling, glycerophospholipid metabolism, steroid hormone biosynthesis, and metabolism of xenobiotics by cytochrome P450. The prominence of these pathways infers their potential relevance to the initiation and progression of OP.

Previous metabolomics investigations on human OP have revealed several potential biomarkers, predominantly centered around lipids and amino acids. In our study, employing LC−MS, we pinpointed 13 upregulated and 8 downregulated metabolites in men, whereas in women, the count was 3 upregulated and 24 downregulated metabolites. Conversely, GC−MS analysis showcased 2 upregulated and 10 downregulated metabolites in males, and 2 upregulated and 1 downregulated metabolite in females. Predominantly, these identified metabolites fall into categories such as lipids and lipid-like molecules, organic acids and derivatives, benzenoids, phenylpropanoids and polyketides, and organic oxygen compounds.

Amino acids (AAs), integral constituents of proteins, assume a crucial role in the regulation of bone metabolism. Nonetheless, the impact of AAs on bone health lacks a definitive consensus, given their potentially divergent effects. In our investigation, osteoporotic men exhibited upregulation of 2-aminomuconic acid semialdehyde and pyroglutamic acid, alongside downregulation of N-acetylornithine and homocysteine. 2-Aminomuconic acid semialdehyde, an intermediary in tryptophan’s oxidative metabolism through the kynurenine pathway in mammals (22), generates oxidation products, such as kynurenine, impeding bone marrow mesenchymal stem cell proliferation. This inhibition may result in compromised osteoblast proliferation and differentiation, contributing to bone loss (15, 23). Refaey et al. (24) similarly demonstrated that the tryptophan byproduct kynurenine, accumulating with age, can induce bone loss. Testosterone may play an important role in regulating Kyn/AhR signaling in musculoskeletal tissues, suggesting that crosstalk between androgenic steroids and Kyn signaling may influence age-related musculoskeletal frailty (25). Pyroglutamic acid, a cyclic nonprotein-derived amino acid formed through glutamine or glutamate deamidation or dehydration, respectively (26), modulates bone metabolism via osteoclasts and is convertible to glutamate (11). Elevated glutamine may lead to bone resorption through glutamate receptor expression on osteoblasts, potentially explaining the association with reduced BMD levels (27). Ornithine, directing the urea cycle, enhances insulin and hormone levels, crucial for muscle building and maintenance during physical activity, countering muscle loss during aging. As protein synthesis efficiency diminishes with age, ornithine’s ability to boost growth hormone levels accelerates muscle tissue production, mitigating age-related effects (28, 29). This could contribute to the observed decrease in plasma N-acetylornithine in OP patients. In conclusion, amino acids have a profound impact on osteogenesis and can induce dysfunctional autophagy and senescence. Moreover, amino acid profiles may differ between genders. Panahi et al.’s study specifically indicates that tryptophan exhibits the most significant negative correlation with osteoporosis in men (30). Metabolomics studies may reveal differences in amino acid metabolism between men and women, possibly related to the effects of gender on protein synthesis and degradation. The intricate interplay between amino acid metabolism, gender-specific factors, and bone health highlights the need for further exploration to unravel the underlying mechanisms.

In our study, the down-regulation of 4-Methoxycinnamic acid, 3,4,5-Trimethoxycinnamic acid, and 3-(3,4,5-Trimethoxyphenyl)propanoic acid was observed in female osteoporosis patients, classifying them within the Phenylpropanoids and polyketides category. This finding prompted an exploration of the potential therapeutic impact of certain phenylpropanoids on bone health. Hydroxycinnamic acid (HCA), originating from cinnamic acid in botanical sources and fruits, demonstrates discernible anabolic effects on bone *in vitro* (31). HCA stimulates osteoblastogenesis and inhibits osteoclastogenesis both *in vitro* and *in vivo*, thereby augmenting bone mass (32). HCA has been identified to induce osteoclastogenesis and suppress osteoclastogenesis through the inhibition of nuclear factor-kappa B (NF-κB) signaling. Conversely, activation of NF-κB signaling by tumor necrosis factor-alpha (TNF-α) or NF-κB ligand receptor agonists implies a molecular mechanism underlying HCA’s anabolic effect on bone (33). NF-κB antagonists have proven effective in mitigating bone loss resulting from estrogen deficiency in murine models (34). HCA, a natural NF-κB antagonist, inhibits NF-κB signaling induced by RANKL in osteoclast precursors and alleviates TNF-α’s inhibitory impact on the anabolism-promoting Smad pathway (35). In conclusion, these investigations robustly support the protective role of Phenylpropanoids and polyketides against osteoporosis, aligning with our findings of down-regulated metabolites in female osteoporosis patients. Importantly, differences in sex hormone levels may influence metabolic pathways and the levels of related metabolites. Postmenopausal osteoporosis, primarily attributed to estrogen deficiency, leads to the production of pro-osteoclastogenic cytokines like TNFα and RANKL. These cytokines notably enhance osteoclast lifespan and activity (36). In women, a significant reduction in estrogen levels induces oxidative stress, potentially contributing to osteoporosis development, while men naturally exhibit lower estrogen levels. This contextualizes the observed metabolic changes in female osteoporosis patients and emphasizes the role of hormonal dynamics in bone health.

Lipid metabolism both processes lipids in the body and provides essential lipids to bone cells, including glycerophospholipids, fatty acyls, steroids, and derivatives (37). In men with osteoporosis, there is an up-regulation of phosphatidylcholine (PC) (25:0/18:0) and PC[O-18:0/O-2:1(1E)], aligning with Mei et al.’s findings of a negative correlation between levels of glycerophospholipid PC and BMD (38). The increased PC content suggests an oxidative stress response, which may lead to bone loss and increased fragility, thereby exacerbating osteoporosis progression (16, 39). Conversely, in female patients with osteoporosis, there is an upregulation of cholic acid (CA) expression, with CA being essential in lipid and cholesterol metabolism. Elevated CA: chenodeoxycholic acid (CDCA) ratio suggests a shift in bile acid metabolism from the traditional to an alternative pathway in osteoporosis patients, consistent with research findings (40). Focusing on fatty acyl groups, tetradecanedioic acid was up-regulated in the female osteoporosis group, mirroring Zhu et al.’s observations of elevated fatty acyl groups in osteoporotic mice bone tissues using UPLC-Q-TOF-MS13 (41). This highlights the significance of disorders in fatty acyl metabolism contributing to osteoporosis. Notably, deficiency of sex hormones, particularly estrogen, plays a crucial role in bone loss in both sexes, with a more pronounced impact on women (38). Postmenopausal women with osteoporosis experience a significant decline in estrogen levels, disrupting the delicate balance between bone remodeling and resorption. Estrogen-related alterations in steroid metabolism emerge as a potential contributing factor to osteoporosis in women, affecting bone density regulation and the balance between bone remodeling and resorption.

Overall, the metabolomic profiles we obtained are promising. These potential biomarkers are biologically important for diagnosis and recurrence monitoring in men and women with OP. (1) We used an advanced high-resolution mass spectrometry metabolomics approach to capture up to 7000 spectral features in human plasma and identify over 1000 metabolites, which is unprecedented in most studies. (2) We used propensity score matching in selecting the OP control population to exclude the effect of confounding factors such as age and BMI. There are some limitations of this study. First, the sample size was small, and the results should be validated in the future in a larger population suffering from OP. Second, this study explored only plasma as the sample, and the results were relatively incomplete. Therefore, more kinds of samples can be selected for OP metabolome study in the future, such as bone marrow and urine, to establish a more complete metabolic database. Third, it failed to provide information on parameters such as hormonal status, IGF1, insulin, vitamin D, and calcitonin in the subjects, and it is important that we take these key factors into account in future studies for a more comprehensive understanding of metabolomic changes associated with BMD.

## 5 Conclusion

Through rigorous data analysis, we observed a predominant association of metabolic markers in men with lipid and amino acid metabolism disorders, while in women, these markers were linked to lipid and Phenylpropanoids and polyketides metabolism. These metabolic shifts influence the bone microenvironment and contribute to systemic homeostatic changes in osteoporotic older adults. Notably, 2-Aminobutyric acid semialdehyde emerges as a promising biomarker for osteoporosis screening in older adults. Future investigations should include extensive validation experiments to substantiate the applicability of our biomarker study results in clinical settings. Repeated validation experiments are imperative to underscore the broad utility of our identified biomarkers in clinical practice.

## Data availability statement

The raw data supporting the conclusions of this article will be made available by the authors, upon reasonable request. Requests to access the datasets should be directed to QG (guoqijp@gmail.com).

## Ethics statement

The studies involving humans were approved by the Shanghai University of Medicine and Health Sciences Ethics Committee. The studies were conducted in accordance with the local legislation, institutional requirements, and the Declaration of Helsinki. The participants provided their written informed consent to participate in this study.

## Author contributions

YHW: Investigation, Writing – original draft, Methodology, Writing – review & editing. CY: Writing – original draft. PH: Methodology, Writing – original draft. JLG: Investigation, Methodology, Writing – review & editing. YW: Investigation, Writing – review & editing. CC: Investigation, Writing – review & editing. CH: Investigation, Writing – review & editing. KZ: Investigation, Writing – review & editing. YQ: Investigation, Writing – review & editing. JL: Investigation, Writing – review & editing. ZX: Investigation, Writing – review & editing. FL: Investigation, Writing – review & editing. DL: Supervision, Writing – review & editing. JG: Supervision, Writing – review & editing. XL: Supervision, Writing – review & editing. QG: Funding acquisition, Supervision, Writing – review & editing.
