# Supplementary material for: Discovery of potential biomarkers for osteoporosis using LC/GC−MS metabolomic methods
Source: Front Endocrinol (Lausanne). 2024 Jan 17;14:1332216. doi: 10.3389/fendo.2023.1332216 (PMC10828954; doi:10.3389/fendo.2023.1332216)

Supplementary Figure 1-A-boxplot

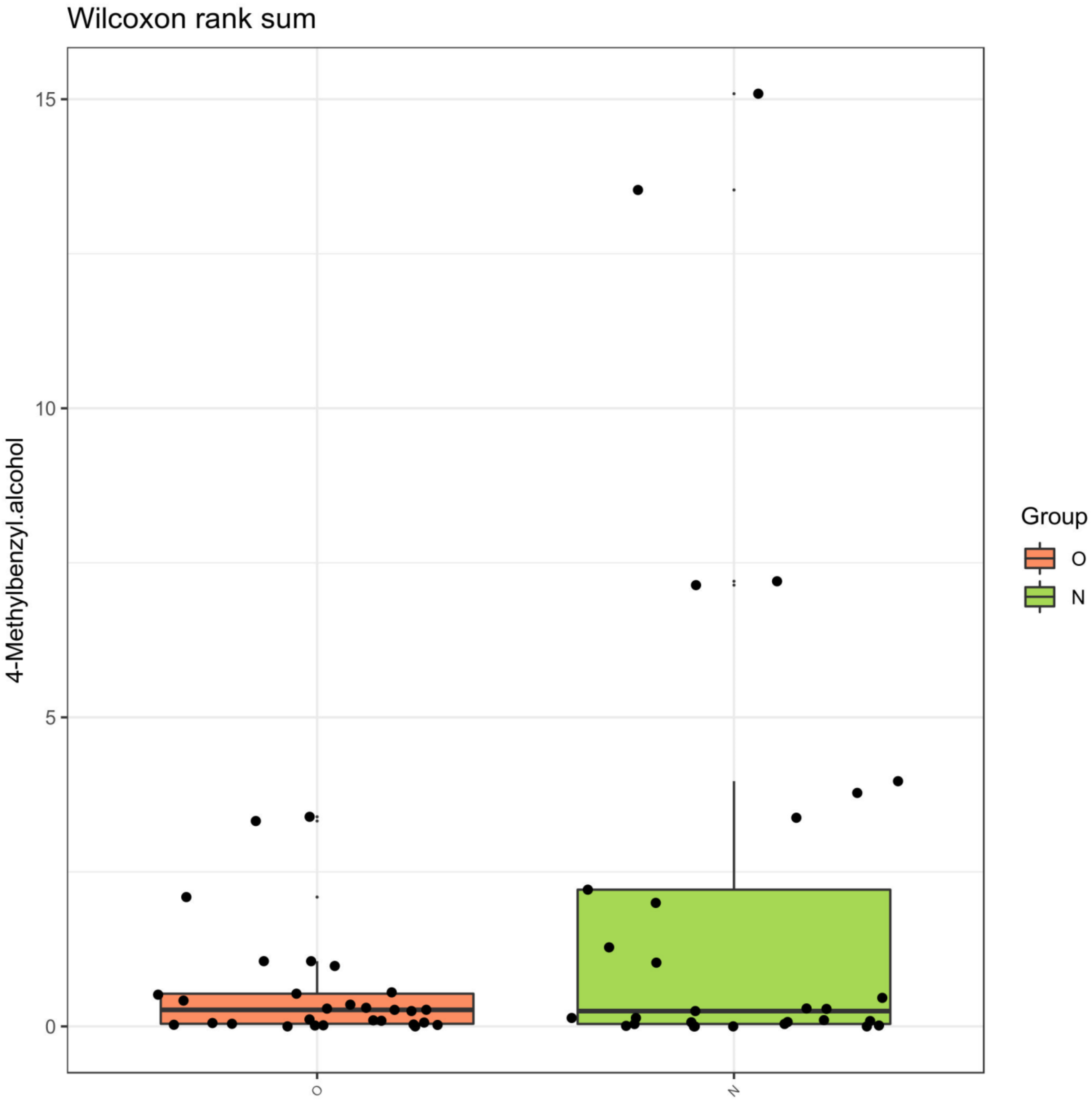

Supplementary Figure 1-B-boxplot

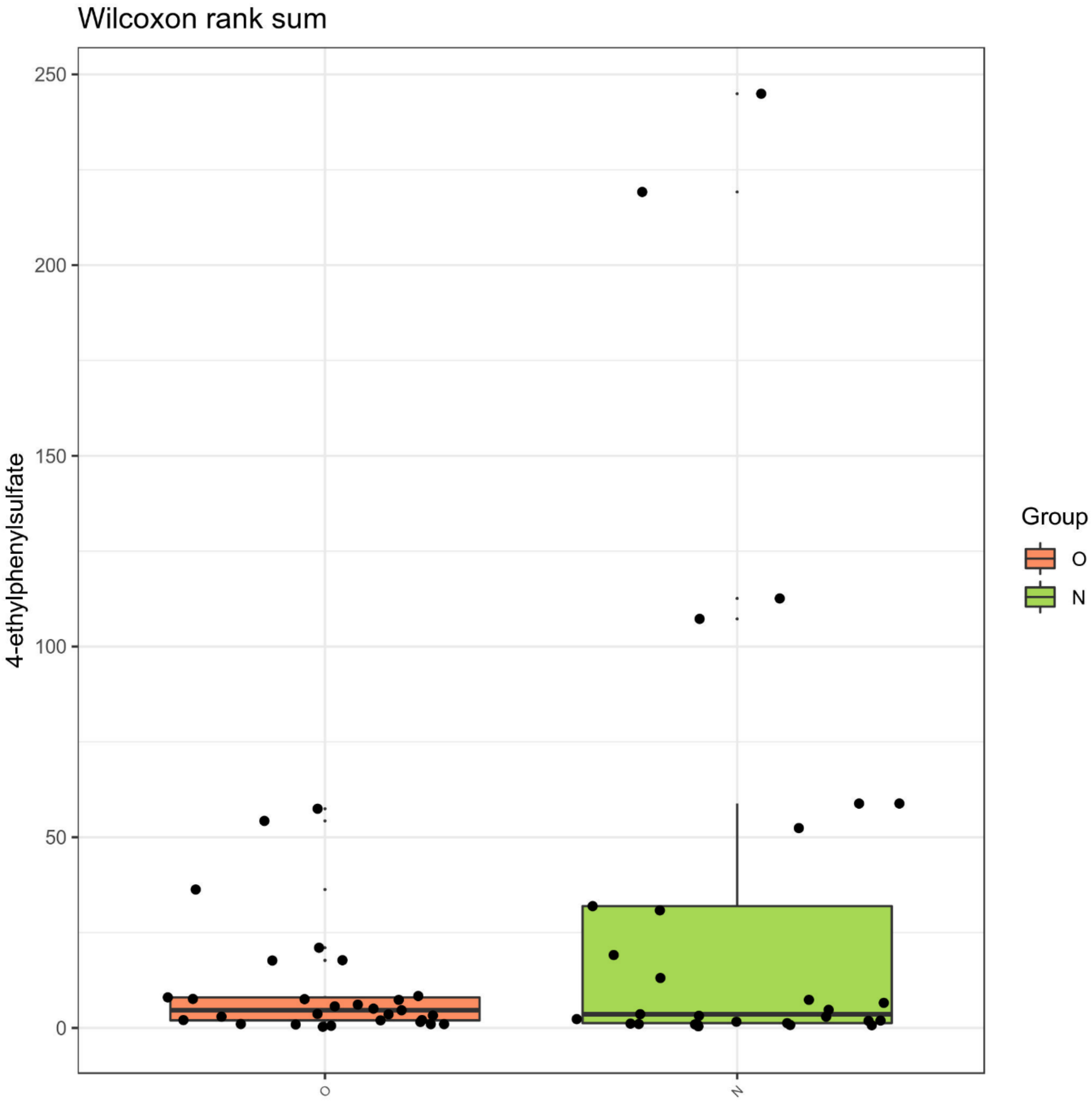

Supplementary Figure 1-C-boxplot

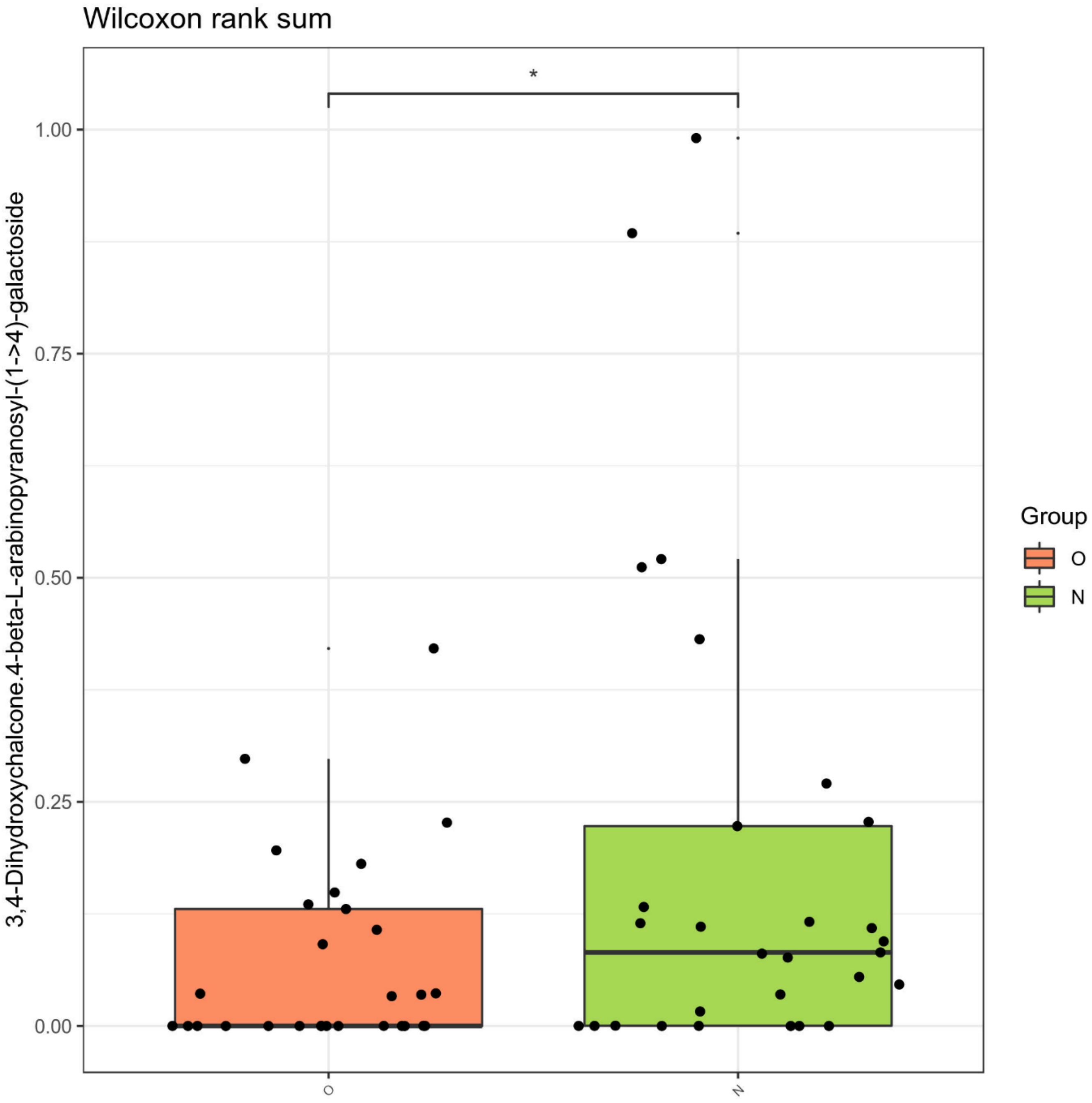

Supplementary Figure 1-D-boxplot

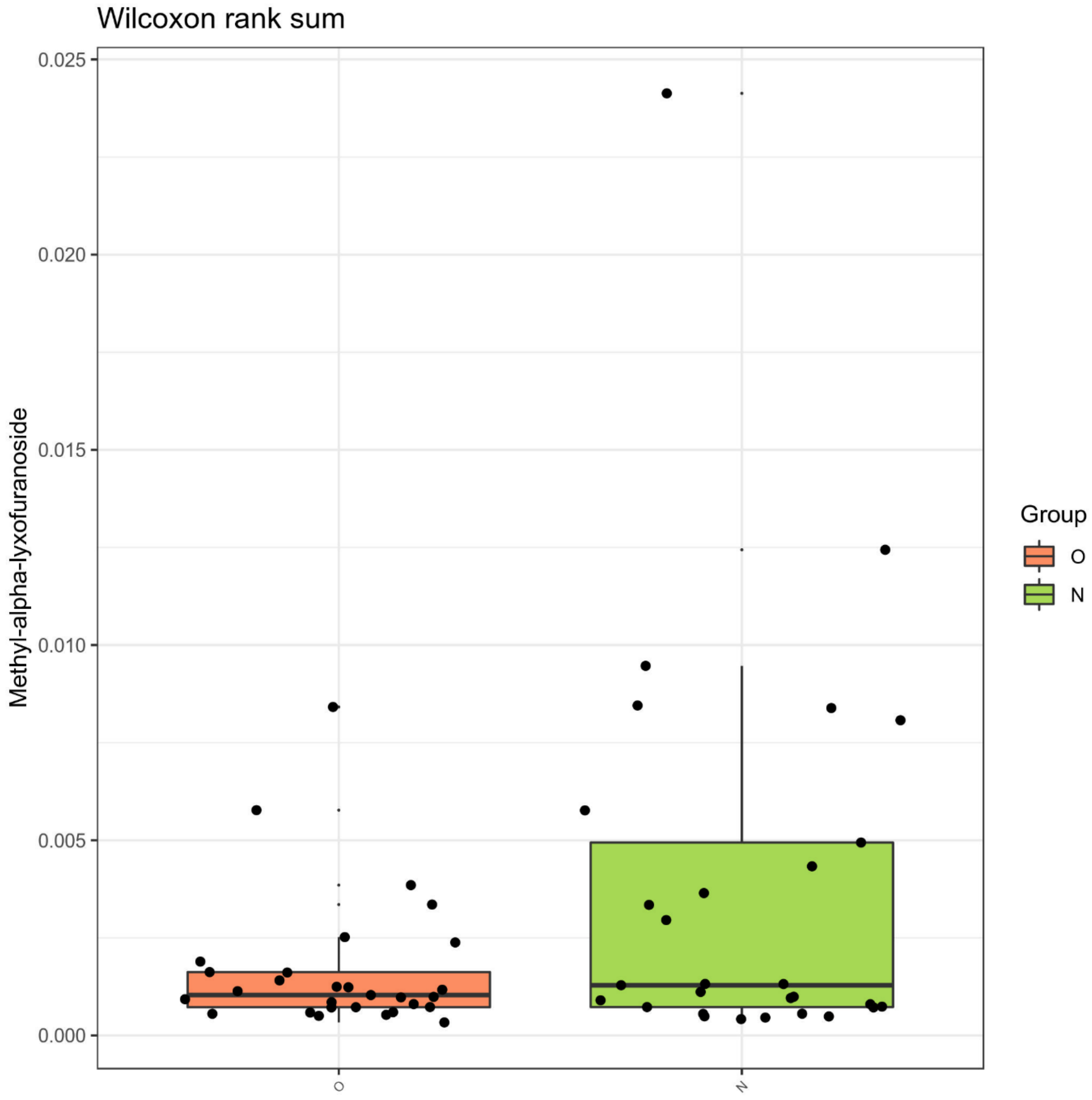

Supplementary Figure 1-E-boxplot

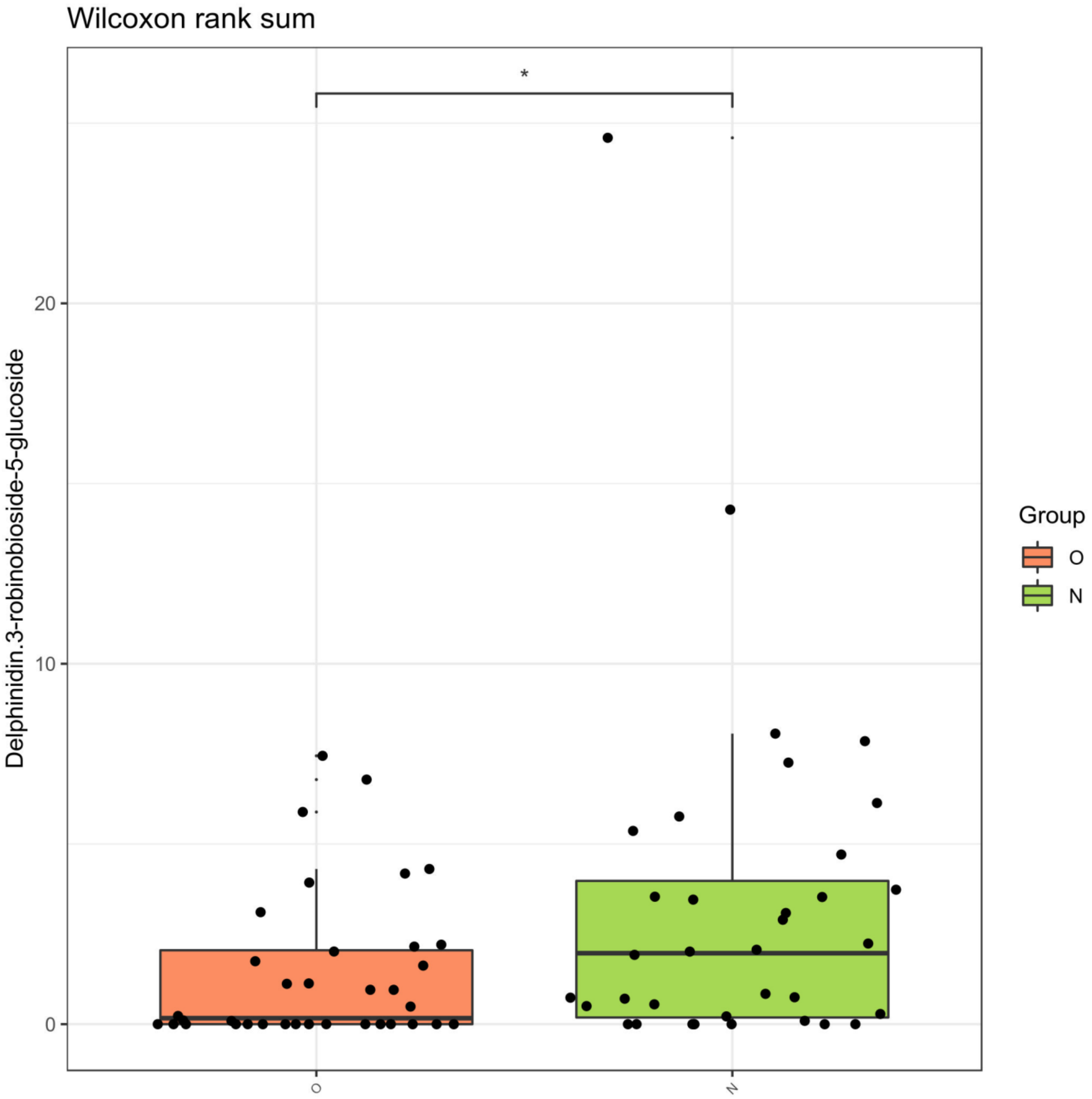

Supplement: Supplementary file 1 [file Image_1.pdf]
